# Mobile Phone App to Promote Lifestyle Change in People at Risk of Type 2 Diabetes: Feasibility 3-Arm Randomized Controlled Trial

**DOI:** 10.2196/63737

**Published:** 2025-01-15

**Authors:** Gyri Skoglund, Gunvor Hilde, Pernille Lunde, Venessa Vera Cruz Naceno, Cecilie Fromholt Olsen, Birgitta Blakstad Nilsson

**Affiliations:** 1 Department of Rehabilitation Science and Health Technology, Faculty of Health Sciences, Oslo Metropolitan University, PB 4, St. Olavs plass, Oslo, NO-0130, Norway, 4747350327; 2 Section for Allied Health Professionals, Department of Neurology, Oslo University Hospital, Oslo, Norway; 3 Section for Physiotherapy, Division of Medicine, Oslo University Hospital, Oslo, Norway

**Keywords:** prevention, risk of type 2 diabetes, lifestyle change, feasibility, app adherence, mobile health, mHealth, mobile phone

## Abstract

**Background:**

The use of mobile health interventions, such as apps, are proposed to meet the challenges faced by preventive health care services due to the increasing prevalence of type 2 diabetes (T2D). Thus, we developed and conducted initial feasibility testing of the Plunde app for promoting and monitoring individual goals related to lifestyle change for people at risk of T2D.

**Objective:**

The primary aim of this study was to assess the feasibility of an app for promoting lifestyle change in people at risk of T2D. The secondary aim was to assess recruitment rate, resource requirements, and change in potential outcomes for a full scale randomized controlled trial (RCT) study .

**Methods:**

A 3-arm feasibility RCT lasting 12 weeks was designed. Participants were recruited from 9 general practitioners in Norway. Eligible participants were randomized to either (1) app follow-up; (2) app follow-up and referral to care as usual in Healthy Life Centers; or (3) referral to care as usual in a Healthy Life Center, only. The primary outcome was feasibility and was measured by app adherence (actual usage of the app), the System Usability Scale, and app motivation score gained from a questionnaire designed for this study. Criteria for success were preset based on these measures. Secondary outcomes included recruitment rate, resource requirements, and potential primary outcomes of a full-scale RCT. This included change in body weight, waist circumference, and self-evaluated functional health status, assessed with the Dartmouth Primary Care Cooperative Research Network/World Organization of Family Doctors (COOP/WONCA) functional health assessment chart.

**Results:**

Within 8 months, 9 general practitioners recruited a total of 54 participants, of which 45 were eligble for participation in the study. Mean age was 61 (SD 13) years and 53% (n=24) were female. App adherence was 86%, the mean System Usability Scale score was 87.3 (SD 11.9), and the mean app motivation score was 74.8 (SD 30.3). Throughout the intervention period, health care professionals spent on average 3.0 (SD 1.0) minutes per participant per week providing follow-up. Statistically significant reduction in body weight and waist circumference was shown in group 1 and 3.

**Conclusions:**

Based on the preset criteria for success, the Plunde app is feasible in providing support for lifestyle change. The Plunde app had excellent user satisfaction. The amount of time spent on monitoring and promoting lifestyle change through the app was low; however, the recruitment was slow. Results from this study will guide the development of further research within this field.

## Introduction

There is an urgent need to prevent and combat the global epidemic of type 2 diabetes (T2D). The number of adults with diabetes is expected to reach 67 million by 2030 and 69 million by 2045 [1]. Prediabetes, an intermediate stage of glucose dysregulation that may precede T2D, affected approximately 720 million individuals worldwide in 2021 and is estimated to affect estimated 1 billion people by 2045 [1,2]. Prediabetes is diagnosed when blood glucose levels are above the normal range but not high enough to be classified as diabetes [2].

Healthy diet, regular physical activity, and maintaining a normal body weight are established cornerstones of prevention or delayed onset of T2D [3-5]. Hence, lifestyle modification is the preferred initial approach when having confirmed prediabetes or being at high risk of T2D [2]. Siginficant risk reduction in delaying or preventing progression into T2D has been found in people receiving lifestyle interventions [3,6]. A post-hoc analysis of randomized, controlled, multicentre Prediabetes Lifestyle Intervention Study showed that a weight loss of 5 percent or more, could decrease the risk of T2D with 72%, and that full remission of T2D should be the target aim [7]. A larger waist circumference (WC), independent of overall adiposity, has been found to be strongly and linearly associated with a higher risk of T2D [8]. Consequently, both body weight and WC may be important predictors of the development for T2D.

Healthy Life Centers (HLCs) is a part of the public health care service in the municipalities in Norway, providing in person lifestyle modification programs for people with, or in high risk of disease [9]. Considering the burden of noncommunicable diseases (NCDs), together with an aging population, the health care system faces fundamental challenges in delivering optimal care [10]. Thus, the primary health care services are at a threshold limit.

The use of mobile health (mHealth) technologies has been proposed to meet challenges related to delivery of sustainable prevention strategies [11]. The beneficial effects of mHealth interventions regarding lifestyle factors have been demonstrated in patients with NCDs in several systematic reviews [12-14]. However, few studies have examined the effect of using an app to deliver support and guidance for risk reduction in patients at risk of T2D.

In 2022, we investigated barriers and facilitators for lifestyle change in people at risk of T2D in a qualitative meta-synthesis [15]. We found that central barriers were time, cost, and availability [15]. Additionally individual tailoring of lifestyle interventions was highlighted to facilitate the process of making healthy changes [15]. The advantages of digital health solutions lie, in its potential to overcome the barriers for lifestyle change and at the same time enabling personalized, empowering, and person-centered health interventions [16]. Therefore, based on former research, user orientation- and involvement, we developed the Plunde app (People Living UNDEr change) to monitor and guide individuals in initiating and maintaining lifestyle change and healthy behavior [17].

Randomized controlled trials (RCTs) are needed to assess potential effect of lifestyle interventions delivered by an app. Before commencing such a trial, investigating the feasibility of using an app to promote lifestyle change is warranted. Therefore, the primary aim of this study was to assess if the Plunde app was feasible to use for promoting lifestyle change in people at risk of T2D. Preset criteria for success was based on app adherence (actual usage of the app), the System Usability Scale (SUS) and app motivation score which was gained from a questionnaire designed for this feasibility study. Secondary, we aimed to assess recruitment rate, resource requirements and change in body weight, WC, and self-evaluated functional health status.

## Methods

### Study Design

This 3-arm feasibility RCT, had an intervention period lasting for 12 weeks. The participants were randomized to receive: lifestyle intervention delivered by the Plunde app (group 1) or referral to group-based lifestyle intervention at the local HLCs in addition to follow-up with the Plunde app (group 2) or to usual care which was referral to group-based lifestyle intervention at the local HLCs (group 3). A computer-generated block randomization scheme was used to allocate the participants to 1 of the 3 groups via concealed envelopes after baseline assessment. The block sizes varied by 3 and 6.

Reporting follows the Consilidated Standard of Reporting Trials (CONSORT) 2010 statement: extension to randomized pilot and feasibility trials [18]. Additionally, principles from the Consilidated Standards of Reporting Trials of Electronic and Mobile Health Applications and Online Telehealth (CONSORT-eHEALTH) statement [19] were applied (Checklist 1).

### Setting and Recruitment

Participants were recruited from general practitioner (GP) clinics in the eastern part of Norway from May 2023 and throughout December 2023. Initially the patients were recruited from 2 GP clinics. From September 2023 we included additionally 3 GPs clinics. Prior to recruitment, information meetings at the clinic sites were held. Eligible participants were asked by their GP regarding willingness to participate in the study, and with their consent they were contacted by a researcher (BBN or VVCN). The baseline assessments and follow-ups were conducted at 3 collaborating HLCs by 2 of the project members (BBN and VVCN).

### Participants

Eligible participants were women and men aged 18 years or older, who were assessed by their GP to be at high risk of developing T2D. The criteria for being at high risk was HbA_1c_ within the threshold level of 42-48 mL/mmol. In addition, participants had to own and use an Android or iOS smartphone and be able to read and understand Norwegian or English.

By September 2023 only 25 participants were included; therefore, we invited 3 more GP clinics to recruit participants to the project. To increase the recruitment rate, we also widened the criteria for being at high risk of T2D. Hypertension, hypercholesteremia, and being obese were set as criteria in addition to, or instead of, HbA_1c_ levels between 42 mL/mmol and 48 mL/mmol.

### Baseline Assessment

At baseline we collected demographic and descriptive data within different lifestyle domains from the standardized “HLC Startup” questionnaire developed by the Norwegian Health Directorate [20]. The demographic data collected included gender, age, educational level, and occupational status. The descriptive data included physical activity level, dietary habits, tobacco, alcohol, sleep habit, and social support. This questionnaire was primarily used to guide and structure the plan for lifestyle change and setting of goals and tasks. Anthropometric data (body weight, WC, and height) were collected at baseline and at follow-up by the same researcher.

### The Plunde App

**Figure 1.**
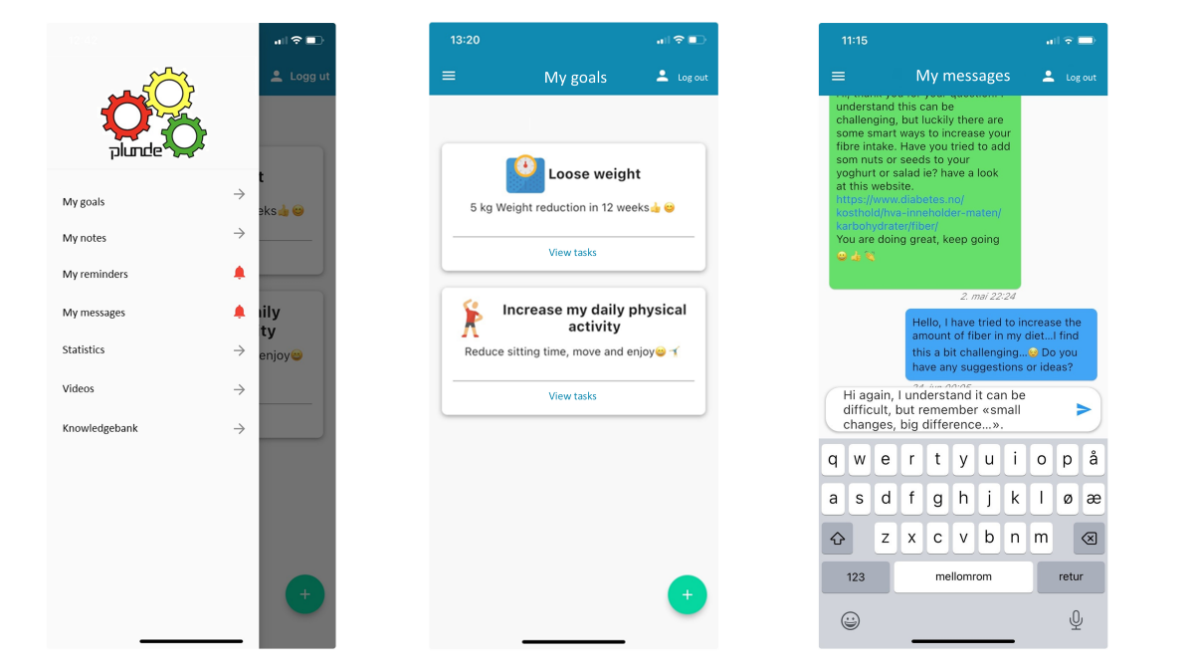
Screenshots showing the user interface.

Participants randomized to the app intervention groups, group 1 and 2, received access to Plunde and guiding in how to use it after baseline assessment. Plunde was developed to promote initiation of behavioral change and adherence to healthy behavior [17]. The app is highly individualized as it is based on the patient’s own goals to initiate or maintain a lifestyle change [17]. Plunde permits the user to set personal goals (Figure 1B) with tasks and accompanying reminders. Goals and tasks decided during the baseline consultation were added to the app. The goals set by the participants were related to the different areas of lifestyle they wanted to work on during the intervention period. The main lifestyle domains presented (through the questionnaire) were dietary habits, physical activity level and exercise, sleep, mental well-being, alcohol, and smoking habits. Each participant set different tasks to their goals, and 1 goal could have 1 or more related tasks. To each of the tasks, the participant set a reminder and decided when and how often the reminders of their tasks should appear. Additionally, they could choose the wording of their reminders. Plunde provided push notifications related to the individual tasks, and the participants replied “completed” or “not completed.”

Other features and functions in Plunde include a note function, messages, videos, and a knowledge bank (Figure 1A). The note function allowed the participant to write relevant notes or comments on their goal achievement process, or for example writing an exercise diary. The message function allowed the participants to chat with their supervisor during the follow-up period (Figure 1C). Relevant videos based on the participants’ goals were added under the video function. In the knowledge bank, the supervisor could add written educational content based on the participants’ goals and requests.

A supervisor had access to an administrator interface and monitored the goals, tasks, and notes of each participant throughout the intervention period. During the follow-up period the supervisor could send weekly encouraging messages to support their process of lifestyle change. If the participants asked any questions in the message function, these were answered within 2 working days. The 2 supervisors in this study were both physiotherapists, 1 being a researcher with comprehensive experience in cardiac rehabilitation and cardiac research and the other with experience from HLCs and community health services. The same supervisor who conducted the baseline consultation monitored and provided feedback throughout the study period.

### Referral to Healthy Lifestyle Center

Participants randomized to group 2 and 3 were referred to the HLC by the respective supervisor immediately after the baseline consultation, with an expected enrollment to the HLC program within 2 weeks. The lifestyle modification program at the HLC included individual and group-based counseling and courses for increased physical activity, healthy diet, improved sleep, and tobacco cessation. The participants were encouraged to attain at least 1 or 2 exercise sessions per week at the HLC. The participants in all the 3 study arms, received counseling based on elements from motivational interviewing, which is a client-centered method of intervention focused on enhancing intrinsic motivation and behavioral change [21]. Goal setting and planning were crucial parts of this conversation, based on the needs, values, and perspectives of the participants.

### Primary Outcome

The primary outcome was feasibility measured as app adherence, user satisfaction, and app motivation. Data related to feasibility of Plunde were collected from patients in groups 1 and 2.

Adherence to the app was registered in terms of actual use, which we defined to be the percentage of reminders answered per week throughout the 12-week intervention period. These data were collected from the administrator interface.

The user satisfaction with the app was assessed with the SUS. The SUS is a technology independent, 10-item questionnaire with a score between 0 and 100, where 0 represents low usability and 100 represent high usability [22]. The elements of the SUS scale cover aspects of usability such as ease of use, integration of functions, learning curve, satisfaction, user confidence, and complexity [22,23].

App motivation was measured with a questionnaire designed for this study. This questionnaire consisted of 17 questions—13 questions with Likert scale (0 to 100) options, 3 multiple-choice questions, and 1 open-ended question (Multimedia Appendix 1). The main questions defining app motivation score was based on 2 of the questions; to what extent Plunde was experienced as motivating and if the individual feedback provided from the supervisor was experienced as motivating. In addition, we asked for how long the participants would have liked to continue using the app and, in this period, how often they would like to receive feedback from their supervisors. The criteria for feasibility success of the app that were preset were (1) at least 80% of the participants reminders were answered per week (app adherence), (2) mean SUS score ≥65, and (3) app motivation score ≥75.

### Secondary Outcomes

To inform a potential full-scale RCT, data on recruitment rate and resource requirements were collected throughout the study period. We calculated the recruitment rate as the number of participants successfully recruited for the study divided by the total amount of time required for recruitment. Resource requirements in groups having the app intervention was assessed by the supervisors who logged all time spent on monitoring and providing feedback to participants. Additionally, potential primary outcomes in a future scale RCT were evaluated. This included body weight, WC, and self-evaluated functional health status. These outcomes were assessed to get experience with the measurements and to examine a potential change.

Body weight was measured in kilogram using a digital scale (Body Composition Analyzer, BC-418, ADE MeWa GMbh, Schwerin and Beurer GS400 Signature Line weight). The evaluation was carried out in the “standard mode,” introducing the participant’s age, sex, and height. We strived to use the same equipment for measuring body weight at baseline and at follow-up. WC was measured with a measuring tape (in cm; Seca 201) at the end of several consecutive natural breaths, midpoint being between the top of the iliac crest and the lower margin of the last palpable rib in the mid axillary line [24]. The cut off points for abdominal obesity and increased risk of T2D is WC ≥80 in women and WC ≥90 in men [25,26].

Self-evaluated functional health status was assessed with the Dartmouth Primary Care Cooperative Research Network/World Organization of Family Doctors (COOP/WONCA) chart [27]. The questionnaire is a generic questionnaire comprising 6 domains—physical fitness, feelings (mental well-being), daily activities, social activities, change in health, and overall health. Each dimension is illustrated pictorially, numerically, and in writing inquiring about the patient’s status during the past 2 weeks. The response categories were scored from 1 to 5, where higher score indicate worse health status.

### Statistical Analysis

Data were analysed using SPSS Statistics for Windows (version 29.0.0; IBM Corp). Descriptive statistics were used to assess feasibility measures. Descriptive statistics are reported in mean and SD or median and range for continuous variables and in number and percentages for categorical variables. To examine a potential change throughout the intervention period for body weight, WC, and self-evaluated functional health status, we performed within-group analyses on mean change from baseline to 12-weeks follow-up. If the data qualified for a normal distribution, the paired sample *t* test was used. If not normally distributed, the paired Wilcoxon signed rank test was used. All statistical tests were 2-sided and *P* values <.05 were considered statistically significant. To ensure a robust sample for the purpose of this feasibility study, we aimed to include a total of 60 participants [18].

### Ethical Considerations

The Regional Committee for Medical Research Ethics in Southeast Norway did not find approval to be required. The study is approved by The Norwegian Data Protection Service for Research and the study has been registered on ClinicalTrials.gov (NCT06117098). The participants provided written informed consent before participation.

## Results

### Recruitment

**Figure 2.**
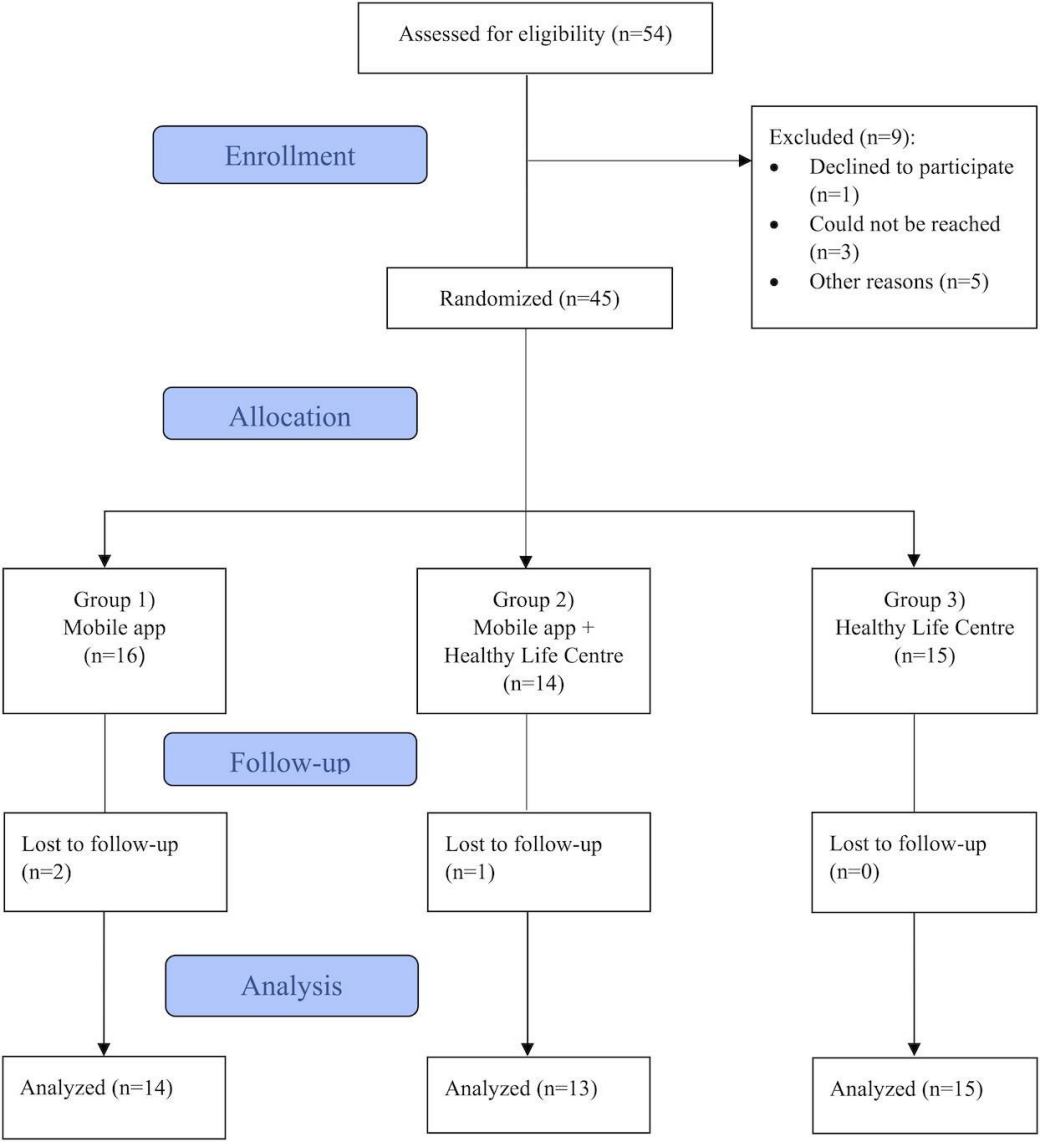
Recruitment and participant flow.

From May 2023 and throughout December 2023, 9 GPs recruited a total of 54 participants of which 45 (82%) were eligible and included in this study. As shown in Figure 2, three participants dropped out during the intervention period. No reasons for drop out were provided.

### Participant Characteristics

Baseline characteristics are presented in Table 1. In total, 53% (n=24) were female and the mean age was 61 (SD 13) years with a range from 31 to 83 years. A total of 49% (n=22) of the participants had BMI ≥30, 88% (n=39) of the women had WC ≥80 cm, and 95% (n=43) of the men had WC ≥90 cm. The goals of the participants pertained to the 5 lifestyle domains of weight reduction, reduced HbA_1c_, healthy diet, physical activity, and sleep.

**Table 1.** Baseline characteristics.

Characteristics	Study sample (N=45)	App (group 1; n=16)	App + Healthy Life Center (group 2; n=14)	Healthy Life Center (group 3; n=15)
Female, n (%)	24 (53)	6 (38)	9 (38)	9 (38)
Age (years), mean (SD)	60.8 (12.6)	54.5 (12.4)	59.1(12.7)	68.1 (9.1)
Body weight (kg), mean (SD)	91.0 (22.3)	96.6 (19.6)	84.6 (24.3)	89.7 (22.8)
BMI, mean (SD)	31.0 (6.6)	32.3 (5.4)	30.0 (7.0)	30.0 (7.0)
Waist circumference (cm), mean (SD)	105.2 (14.9)	108.5 (12.1)	99.0 (16.0)	106.7 (15.4)
**Educational level, n (%)**				
Elementary or high school	20 (46)	7 (44)	7 (50)	7 (47)
University or college >3 years	9 (20)	3 (19)	2 (14)	4 (27)
University or college ≤3 years	14 (33)	5 (31)	5 (36)	4 (27)
**Employment, n (%)**				
Full-time work or part-time work	19 (43)	9 (56)	8 (57)	4 (27)
Disability benefits	8 (18)	5 (31)	0 (0)	3 (20)
Retired	17 (39)	2 (13)	6 (43)	8 (53)
**COOP/WONCA^a^ score, mean (SD)**				
Physical fitness	2.3 (1.0)	2.0 (0.9)	2.2 (1.1)	2.8 (1.0)
Feelings	1.8 (1.2)	1.8 (1.1)	1.8 (1.1)	1.8 (1.3)
Daily activities	1.5 (0.9)	1.4 (0.7)	1.5 (0.9)	1.7 (1.1)
Social activities	1.4 (0.8)	1.4 (0.8)	1.1 (0.3)	1.7 (1.0)
Change in health	2.6 (0.7)	2.5 (0.6)	2.6 (0.6)	2.8 (0.7)
Overall health	2.4 (0.8)	2.5 (0.7)	2.2 (0.8)	2.5 (0.7)
Total score, mean (SD)	11.8 (3.2)	11.6 (2.8)	10.6 (3.3)	13.1 (3.0)

a COOP/WONCA: Dartmouth Primary Care Cooperative Research Network/World Organization of Family Doctors functional health assessment chart.

Participants randomized to Plunde (groups 1 and 2; n=27) had a mean of 1.5 (SD 0.7) goals, 1‐3 tasks related to each goal and a mean of 7.7 (SD 7.1) weekly reminders of their goal related tasks. The most frequent goal was related to weight reduction and reduced HbA_1c_. Further, 17 (61%) participants randomized to Plunde had iOS smartphone and 11 (39%) had Android smartphone. For 2 (7%) participants, data regarding the operative system were not collected. No technical problems or bugs was reported during the intervention period.

### Primary Outcome

At the 12 weeks follow-up, user log data could be retrieved from 27 participants. The overall app adherence score was 86% (n=27) for the whole intervention period. The mean SUS score was 86.6 (SD 12.0), indicating a grade A and excellent usability (n=22).

The mean app motivation score was 74.8 (SD 30.3). The motivational messaging from the supervisor was reported (n=26) to be highly motivating, with a mean score of 93.5 (SD 19.8). When asked for how long the participants would prefer to be followed up through the Plunde app, the most frequent answer was 12 months. On the question on how often the participants would find it useful to get feedback from their supervisor, every other week was the most frequent answer.

### Secondary Outcomes

From May 2023 and throughout December 2023, 54 participants were recruited from 9 GP clinics within 8 months, of these 45 participants were eligible for the study. The recruitment rate was 1.4 participants per week.

The first consultation with the participants in group 1 and 2 (n=27) lasted in average for 91.3 (SD 26.5) minutes. Monitoring and follow-up through the app during the study period, took an average of 3.0 (SD 1.0) minutes per participant per week.

As presented in Table 2, preliminary pre-post interventions findings showed statistically significant within-group changes in 1 or more outcomes in all 3 groups. A significant reduction in body weight and WC was shown in group 1 and 3, and improved changes in self-evaluated health was shown in all 3 groups. In the data analysis related to weight reduction, there was 1 data point missing from group 1. For WC, 1 data point was missing from both group 1 and 2. Meanwhile, for the COOP/WONCA analysis, group 3 had 1 missing data point.

**Table 2.** Changes in body weight, waist circumference, and self-evaluated functional health status at baseline (N=45) and after 12 weeks follow-up (n=42).

Outcomes and measures	Baseline, mean (SD)	12 weeks, mean (SD)	Mean difference (95% CI)	*P* value
**App (group 1; n=14)**				
Body weight (kg)	96.7 (20.1)	93.8 (18.5)	2.8 (0.8 to 4.9)	.011
Waist circumference (cm)	108.9 (12.4)	104.3 (11.3)	4.6 (2.0 to 7.1)	.002
COOP/WONCA^a^ total score	11.9 (2.8)	9.4 (1.9)	2.5 (1.0 to 4.0)	.003
Physical fitness	2.1 (0.9)	1.8 (0.7)	0.4 (−0.2 to 0.9)	.174
Feelings	1.9 (1.1)	1.5 (0.9)	0.4 (−0.2 to 1.0)	.139
Daily activities	1.4 (0.8)	1.3 (0.7)	0.1 (−0.4 to 0.6)	.547
Social activities	1.4 (0.9)	1.0 (0.0)	0.4 (−0.6 to 0.9)	.082
Change in health	2.5 (0.7)	1.6 (0.6)	0.9 (0.5 to 1.2)	<.001
Overall health	2.6 (0.8)	2.1 (0.8)	0.4 (−0.1 to 0.9)	.082
**App + Healthy Life Center (group 2; n=13)**				
Body weight	81.5 (22.3)	80.2 (21.5)	1.3 (−0.5 to 3.1)	.136
Waist circumference (cm)	98.1 (13.8)	95.5 (15.3)	2.6 (−0.7 to 6.0)	.114
COOP/WONCA total score	10.3 (3.2)	9.4 (2.3)	0.9 (−1.5 to 3.3)	.423
Physical fitness	2.1 (1.0)	1.8 (0.8)	0.3 (−0.1 to 0.7)	.104
Feelings	1.8 (1.2)	1.8 (1.1)	0.0 (−0.7 to 0.7)	>.99
Daily activities	1.5 (1.0)	1.1 (0.3)	0.4 (−0.2 to 1.0)	.209
Social activities	1.1 (0.3)	1.1 (0.3)	—^b^	—
Change in health	2.5 (0.7)	2.2 (0.9)	0.9 (−0.3 to 1.5)	.004
Overall health	2.2 (0.8)	1.6 (0.7)	0.5 (0.1 to 0.9)	.012
**Healthy Life Center (group 3; n=15)**				
Body weight (kg)	89.7 (22.8)	86.9 (21.4)	2.7 (0.9 to 4.6)	.007
Waist circumference (cm)	106.7 (15.4)	103.2 (13.9)	3.5 (1.1 to 6.0)	.009
COOP/WONCA total score	13.1 (3.1)	11.4 (3.3)	1.8 (-0.2 to 3.7)	.070
Physical fitness	2.8 (1.0)	2.4 (1.1)	0.4 (−0.3 to 1.1)	.233
Feelings	1.8 (1.3)	2.1 (1.5)	−0.3 (−0.9 to 0.3)	.265
Daily activities	1.8 (1.1)	1.4 (0.9)	0.4 (−0.4 to 1.1)	.336
Social activities	1.7 (1.1)	1.1 (0.4)	0 (−0.02 to 1.2)	.055
Change in health	2.8 (0.7)	2.1 (0.8)	0.6 (−0.02 to 1.3)	.057
Overall health	2.5 (0.8)	2.0 (0.7)	0.5 (0.1 to 0.9)	.029

a COOP/WONCA: Dartmouth Primary Care Cooperative Research Network/World Organization of Family Doctors functional health assessment chart.

b The paired sample effect size could not be produced.

## Discussion

### Principal Findings

According to our preset criteria for feasibility, we found the Plunde app to be feasible to use for promoting and monitoring lifestyle change for people at risk of T2D. App adherence was high, and Plunde was rated with excellent user satisfaction. Furthermore, the app was evaluated as motivating, and feedback from the supervisors was scored as the most motivating factor. The amount of time spent on monitoring and promoting lifestyle change through the app was low. However, the recruitment was time consuming.

To our knowledge this is the first study assessing the feasibility of a mobile phone app to promote and monitor lifestyle change in people at risk of T2D. In our study, the adherence to the app was high. This is an important finding as the effectiveness of mHealth interventions have been proven to be closely related to app adherence and engagement [28]. Recently, Jakob et al [28] found that 5 intervention-related factors indicated positive effects on adherence to mHealth apps for prevention or management of NCDs. This included tailoring and personalization to the individual needs of the user, reminders in the format of individualized push notifications, being user-friendly, a technically stable app design, and personal support that complemented the digital intervention [28]. In line with this, 2 systematic reviews by Dugas et al [29] and Asbjørnsen et al [30], showed that the most frequent behavior change techniques used in mHealth interventions were goal-setting and planning, personalization, feedback and monitoring as well as prompts and cues. All these factors were integrated in the Plunde app and could, therefore, have contributed to the high adherence to the app. The integration of digital features and behavior change techniques may optimize T2D prevention interventions to achieve clinically significant weight loss [31] and improve self-management [32].

The app motivation scores in our study were high. Particularly, participants had high scores on the motivational feedback from the supervisor. Having “a person behind the app” can also be aligned with a so-called blended care model, characterized by a combination of digital interventions and therapist-guided interventions [33-35]. Blended care intervention has shown promising results on promoting lifestyle change in terms of weight loss and physical activity [34-36]. In previous research, the communication with the supervisor was highlighted as crucial, because of the possibility to communicate and receive individualized feedback by a real person [37]. On the contrary, the lack of direct contact and involvement with health care professionals has in previous study been pointed out as a main barrier for feasibility of an app-based intervention for cardiovascular disease and diabetes risk awareness and prevention [38]. An important component of this form of blended care model is the “onboarding” phase of the app intervention [39]. This is likely to be another factor having contributed to the high app adherence in this study. The initial face-to-face consultation enabled further tailored communication through the app, as it focused on the individual’s goals and tasks, and the associated barriers and facilitators to reach them. The freedom of choice and flexibility in a tailored program allows participants to set personalized and meaningful goals [15]. A systematic review and meta-analysis by Joiner et al [12] showed that technology combined with online health coaching resulted in greater weight loss compared to fully automated electronic health interventions. Thus, it is reasonable to assume that a crucial factor for adherence to the app is the individualized follow-up provided by a supervisor. Consequently, to succeed in a full-scale RCT, each participant should have a supervisor which can serve as the person behind the app and provide personalized and tailored feedback.

Weight reduction is the most important factor in preventing T2D, delaying the onset of T2D, and even remission of prediabetes and micro- and macrovascular risk [6,7]. In this feasibility study we assessed body weight, WC, and self-evaluated health at baseline and at 12 weeks follow-up. This was done to get experience with the measurements and to examine a potential change in these outcomes for a potential full-scale trial. In such a trial, one could consider to include the outcome waist-to-height ratio, as this variable has been shown to have an even stronger association with T2D risk than BMI, WC, and the waist-to-hip ratio [25,40,41]. Importantly, this study was designed as a feasibility RCT and did not aim to detect possible differences between groups, thus between-group analysis was not performed. We got valuable experiences with conducting the measurements and collecting data on body weight and WC.

### Strengths and Limitations

A strength of this study is the heterogeneity of our sample in terms of age, gender, educational level, and sociodemographic status [42]. This increases the reliability and generalizability of our results, particularly regarding app adherence, app motivation, and user satisfaction. However, participation bias can be considered as a limitation in this study, as the people who agreed to participate are more likely to have a higher motivation for lifestyle change and being more aware of their risk of T2D. It is also important to consider that there is a potential risk in failing to recruit people at the highest risk [43]. However, considering the baseline characteristics of our study sample we seem to have reached the targeted population for risk reduction of T2D.

An important limitation to this study is that we changed our inclusion criteria during the recruitment process, jeopardizing a correct picture of the recruitment process. However, widening the inclusion criteria was a pragmatic choice, to achieve an adequate sample for being able to evaluate the feasibility of Plunde. If we had used the widened criteria from the beginning, the recruitment rate could have been faster. Thus, this has given us valuable considerations to the recruitment process in a potential full-scale RCT.

According to the recommendations for complex interventions and assessing feasibility [44] mixed methods is recommended [45]. Thus, not having qualitative interviews at the follow-up after ended intervention may present a limitation. Qualitative interviews could have provided us with valuable and more extensive information to better understand the complexity of adherence and engagement in use of the app. However, researchers from our group have previously investigated participants experiences with app-based follow-up over a period of 1 year [37]. Qualitative data and results from their work prepared the ground when designing the Plunde app in terms of functionality and likewise the role of the app supervisor to promote adherence and engagement to the app [17].

### From a Feasibility Study to a Full-Scale RCT Study

Despite the overall feasibility of the Plunde, our study revealed some adjustments to be made prior commencing a full-scale RCT. To address the challenge of the slow recruitment rate, involvement of a substantial number of GP clinics seems necessary. However, it is important to consider that people at risk of T2D, do not necessarily visit their GP very often, being in a state often without clinical symptoms [2].

Several recruitment strategies could be considered to enhance the recruitment rate and to increase the number of people available for recruitment. This could be done by developing a campaign for recruitment of participant into a full RCT, mediated or passive recruitment by using a wide variety of social media platforms. In person recruitment of people at risk of T2D has been conducted in venues like public spaces, libraries, churches, and different meeting places to reach people assessable for eligibility [46]. Rapid risk assessments and point-of-care testing of HbA_1c_ levels in community settings, for example GP offices, has been suggested to increase access to testing, facilitating early diagnosis, glycemic awareness, and lifestyle change [47], and could potentially increase the recruitment rate. Furthermore, using electronic health record-supported recruitment approaches has been found to be feasible and promising, and have the potential to increase the reach of eligible participants [48]. This could also be an important approach to facilitate the engagement of GPs and health care professionals in the research [48].

In terms of conducting a potential future 3-arm full-scale RCT, this feasibility study has provided us with valuable insights to the final decision of the design. We got experience with the administration of 3 intervention groups, including acceptability of the randomization procedure, and importantly, we gained important experience on the overall recruitment rate. In terms of conducting a potential future 3-arm full-scale RCT, we consider it as a strength to have evaluated feasibility of the app when used as “stand alone” intervention (group 1) and when used in combination with face-to-face group intervention (group 2). However, the complexity and resource requirements required to conduct a 3-arm design in a full-scale RCT must be taken under consideration. Regarding the usual care group (group 3), the variation in modalities offered by the HLCs and the risk of prolonged inclusion time to the HLC program, due to capacity and resource challenges, adds to the complexity and underscores the importance of an integrated stakeholder involvement in the planning and implementation of a full-scale RCT.

An advantage of conducting a full scale RCT with a 3-arm design, would be the potential to investigate whether an app intervention alone is as good as and not inferior to usual care (HLC). In addition, an evaluation whether a lifestyle intervention delivered as combination of app and HLC would be superior to usual care alone. Follow-up through an app instead of participating in HLC could be the preferred alternative for people because of the flexibility and accessibility an app provides. As a significant proportion of the targeted population for risk reduction of T2D are people committed to work and family responsibilities, and in terms of finding optimal prevention strategies, it is a challenge that the HLCs for the most offer their program during daytime and main working hours.

Despite the growing evidence on the effectiveness of mHealth interventions and lifestyle change for risk reduction of T2D, more evidence is needed regarding long-term adherence and effectiveness [28,49,50]. Considering our positive findings on the low amount of time spent on monitoring and providing follow-up, an assessment of long-term adherence and effectiveness implemented in a full-scale RCT would be feasible and sustainable in terms of resource requirements. When we asked the participants in this study how long they would have continued to use the app, most of the participants answered 12 months. In terms of individualized feedback from the supervisor, most of the app participants answered that they found feedback every month appropriate, suggesting that long-term follow does not necessarily need to be time-consuming from a health care professionals’ perspective.

The high speed of technology, innovative app development and the potential of artificial intelligence chatbots as supervisors provides a huge potential in providing scalable mHealth interventions for people at risk of T2D. The need for more research to understand which interventions and components have the greatest reach, promotes engagement and adherence and being most effective in the long term is consistently elucidated [50]. However, this feasibility study leads us to suggest that the potential of digital interventions or mHealth is also to be found beyond the technology itself. The blended care model design of the Plunde app contributes to the research on finding the optimal interaction model between health care professionals and people at risk of T2D.

### Conclusions

Based on our preset criteria for success the Plunde app is feasible to monitor and promote lifestyle change in patients at risk of T2D. The amount of time spent on monitoring and promoting lifestyle change through the app was low; however, recruitment was slow. This feasibility study has provided important information and guidance for planning and executing a potential randomized controlled trial.

## Supplementary material

10.2196/63737 Multimedia Appendix 1 Questions related to the experience with the setup and use of the smartphone application Plunde.

10.2196/63737 Checklist 1 CONSORT-eHEALTH checklist (V1.6.1).

## Abbreviations

CONSORT: Consilidated Standard of Reporting Trials
CONSORT-eHEALTH: Consilidated Standards of Reporting Trials of Electronic and Mobile Health Applications and Online Telehealth
GP: general practitioner
HLC: Healthy Life Center
mHealth: mobile health
NCD: noncommunicable disease
RCT: randomized controlled trial
SUS: System Usability Scale
T2D: type 2 diabetes
WC: waist circumference

## References

[1] Sun H, Saeedi P, Karuranga S (2022). IDF Diabetes Atlas: global, regional and country-level diabetes prevalence estimates for 2021 and projections for 2045. Diabetes Res Clin Pract.

[2] Echouffo-Tcheugui JB, Perreault L, Ji L, Dagogo-Jack S (2023). Diagnosis and management of prediabetes: a review. JAMA.

[3] Magkos F, Hjorth MF, Astrup A (2020). Diet and exercise in the prevention and treatment of type 2 diabetes mellitus. Nat Rev Endocrinol.

[4] Kim HJ, Kwon O (2024). Nutrition and exercise: cornerstones of health with emphasis on obesity and type 2 diabetes management-a narrative review. Obes Rev.

[5] Uusitupa M, Khan TA, Viguiliouk E (2019). Prevention of type 2 diabetes by lifestyle changes: a systematic review and meta-analysis. Nutrients.

[6] Glechner A, Keuchel L, Affengruber L (2018). Effects of lifestyle changes on adults with prediabetes: a systematic review and meta-analysis. Prim Care Diabetes.

[7] Sandforth A, von Schwartzenberg RJ, Arreola EV (2023). Mechanisms of weight loss-induced remission in people with prediabetes: a post-hoc analysis of the randomised, controlled, multicentre Prediabetes Lifestyle Intervention Study (PLIS). Lancet Diabetes Endocrinol.

[8] Jayedi A, Soltani S, Motlagh SZT (2022). Anthropometric and adiposity indicators and risk of type 2 diabetes: systematic review and dose-response meta-analysis of cohort studies. BMJ.

[9] (2017). Healthy Life Centres in Norway. Helsedirektoratet.

[10] Tromp J, Jindal D, Redfern J (2022). World Heart Federation roadmap for digital health in cardiology. Glob Heart.

[11] (2011). mHealth: New Horizons for Health through Mobile Technologies.

[12] Joiner KL, Nam S, Whittemore R (2017). Lifestyle interventions based on the diabetes prevention program delivered via eHealth: a systematic review and meta-analysis. Prev Med.

[13] Lunde P, Nilsson BB, Bergland A, Kværner KJ, Bye A (2018). The effectiveness of smartphone apps for lifestyle improvement in noncommunicable diseases: systematic review and meta-analyses. J Med Internet Res.

[14] Barengo NC, Diaz Valencia PA, Apolina LM (2022). Mobile health technology in the primary prevention of type 2 diabetes: a systematic review. Curr Diab Rep.

[15] Skoglund G, Nilsson BB, Olsen CF, Bergland A, Hilde G (2022). Facilitators and barriers for lifestyle change in people with prediabetes: a meta-synthesis of qualitative studies. BMC Public Health.

[16] Zangger G, Bricca A, Liaghat B (2023). Benefits and harms of digital health interventions promoting physical activity in people with chronic conditions: systematic review and meta-analysis. J Med Internet Res.

[17] Lunde P, Skoglund G, Olsen CF, Hilde G, Bong WK, Nilsson BB (2023). Think aloud testing of a smartphone app for lifestyle change among persons at risk of type 2 diabetes: usability study. JMIR Hum Factors.

[18] Eldridge SM, Chan CL, Campbell MJ (2016). CONSORT 2010 statement: extension to randomised pilot and feasibility trials. BMJ.

[19] Eysenbach G, CONSORT-EHEALTH Group (2011). CONSORT-EHEALTH: improving and standardizing evaluation reports of web-based and mobile health interventions. J Med Internet Res.

[20] (2023). Tilbud ved frisklivssentraler og veilederkurs [Article in Norwegian]. Helsedirektoratet.

[21] Armstrong MJ, Mottershead TA, Ronksley PE, Sigal RJ, Campbell TS, Hemmelgarn BR (2011). Motivational interviewing to improve weight loss in overweight and/or obese patients: a systematic review and meta-analysis of randomized controlled trials. Obes Rev.

[22] Brooke J, Jordan PW, McClelland IL, Weerdmeester B (1996). Usability Evaluation In Industry.

[23] Hyzy M, Bond R, Mulvenna M (2022). System Usability Scale benchmarking for digital health apps: meta-analysis. JMIR Mhealth Uhealth.

[24] (2008). Waist circumference and waist-hip ratio: report of a WHO expert consultation, Geneva, 8-11 December 2008. World Health Organization Institutional Repository for Information Sharing.

[25] Ke JF, Wang JW, Lu JX, Zhang ZH, Liu Y, Li LX (2022). Waist-to-height ratio has a stronger association with cardiovascular risks than waist circumference, waist-hip ratio and body mass index in type 2 diabetes. Diabetes Res Clin Pract.

[26] Siren R, Eriksson JG, Vanhanen H (2012). Waist circumference a good indicator of future risk for type 2 diabetes and cardiovascular disease. BMC Public Health.

[27] Kinnersley P, Peters T, Stott N (1994). Measuring functional health status in primary care using the COOP-WONCA charts: acceptability, range of scores, construct validity, reliability and sensitivity to change. Br J Gen Pract.

[28] Jakob R, Harperink S, Rudolf AM (2022). Factors influencing adherence to mHealth apps for prevention or management of noncommunicable diseases: systematic review. J Med Internet Res.

[29] Dugas M, Gao GG, Agarwal R (2020). Unpacking mHealth interventions: a systematic review of behavior change techniques used in randomized controlled trials assessing mHealth effectiveness. Dig Health.

[30] Asbjørnsen RA, Smedsrød ML, Solberg Nes L (2019). Persuasive system design principles and behavior change techniques to stimulate motivation and adherence in electronic health interventions to support weight loss maintenance: scoping review. J Med Internet Res.

[31] Van Rhoon L, Byrne M, Morrissey E, Murphy J, McSharry J (2020). A systematic review of the behaviour change techniques and digital features in technology-driven type 2 diabetes prevention interventions. D Health.

[32] El-Gayar O, Ofori M, Nawar N (2021). On the efficacy of behavior change techniques in mHealth for self-management of diabetes: a meta-analysis. J Biomed Inform.

[33] Obro LF, Heiselberg K, Krogh PG (2021). Combining mHealth and health-coaching for improving self-management in chronic care. A scoping review. Patient Educ Couns.

[34] Berry MP, Sala M, Abber SR, Forman EM (2021). Incorporating automated digital interventions into coach-delivered weight loss treatment: a meta-analysis. Health Psychol.

[35] Hohberg V, Fuchs R, Gerber M, Künzler D, Paganini S, Faude O (2022). Blended care interventions to promote physical activity: a systematic review of randomized controlled trials. Sports Med Open.

[36] Kouwenhoven-Pasmooij TA, Robroek SJW, Kraaijenhagen RA (2018). Effectiveness of the blended-care lifestyle intervention “PerfectFit”: a cluster randomised trial in employees at risk for cardiovascular diseases. BMC Public Health.

[37] Lunde P, Bye A, Bruusgaard KA, Hellem E, Nilsson BB (2022). Patients’ experiences of using a smartphone app after cardiac rehabilitation: qualitative study. JMIR Hum Factors.

[38] Buss VH, Varnfield M, Harris M, Barr M (2022). Remotely conducted app-based intervention for cardiovascular disease and diabetes risk awareness and prevention: single-group feasibility trial. JMIR Hum Factors.

[39] Bostrøm K, Børøsund E, Varsi C (2020). Digital self-management in support of patients living with chronic pain: feasibility pilot study. JMIR Form Res.

[40] Moosaie F, Fatemi Abhari SM, Deravi N (2021). Waist-to-height ratio is a more accurate tool for predicting hypertension than waist-to-hip circumference and BMI in patients with type 2 diabetes: a prospective study. Front Public Health.

[41] Chen N, Hu LK, Sun Y (2023). Associations of waist-to-height ratio with the incidence of type 2 diabetes and mediation analysis: two independent cohort studies. Obes Res Clin Pract.

[42] Kontochristopoulou AM, Karatzi K, Karaglani E (2022). Sociodemographic, anthropometric, and lifestyle correlates of prediabetes and type 2 diabetes in europe: the Feel4Diabetes study. Nutr Metab Cardiovasc Dis.

[43] Bayley A, Stahl D, Ashworth M (2018). Response bias to a randomised controlled trial of a lifestyle intervention in people at high risk of cardiovascular disease: a cross-sectional analysis. BMC Public Health.

[44] Craig P, Dieppe P, Macintyre S (2008). Developing and evaluating complex interventions: the new Medical Research Council guidance. BMJ.

[45] Skivington K, Matthews L, Simpson SA (2021). A new framework for developing and evaluating complex interventions: update of Medical Research Council guidance. BMJ.

[46] Paglialunga S, Bond R, Jaycox SH (2018). Evaluation of HbA1c screening during outreach events for prediabetes subject recruitment for clinical research. Trials.

[47] Gourlay A, Sutherland C, Radley A (2024). Point-of-care testing of HbA_1_c levels in community settings for people with established diabetes or people at risk of developing diabetes: a systematic review and meta-analysis. Prim Care Diabetes.

[48] Aroda VR, Sheehan PR, Vickery EM (2019). Establishing an electronic health record-supported approach for outreach to and recruitment of persons at high risk of type 2 diabetes in clinical trials: the vitamin D and type 2 diabetes (D2d) study experience. Clin Trials.

[49] Mönninghoff A, Kramer JN, Hess AJ (2021). Long-term effectiveness of mHealth physical activity interventions: systematic review and meta-analysis of randomized controlled trials. J Med Internet Res.

[50] Stowell M, Dobson R, Garner K, Baig M, Nehren N, Whittaker R (2024). Digital interventions for self-management of prediabetes: a scoping review. PLoS One.

