# Supplementary material for: Mobile Phone App to Promote Lifestyle Change in People at Risk of Type 2 Diabetes: Feasibility 3-Arm Randomized Controlled Trial
Source: JMIR Form Res. 2025 Jan 15;9:e63737. doi: 10.2196/63737 (PMC11788870; doi:10.2196/63737)
Supplement: Multimedia Appendix 1 [file formative-v9-e63737-s001.pdf]

Multimedia Appendix 2. Questions related to your experience with the setup and use of the smartphone application Plunde.

Based on your experience with Plunde, please rate each statement with a number from 0 to 100. The number can be consider a percentage; as an example the number 100 equals a 100% correct statement in your opinion.

	**To what extent do you experience that …**	**Numeric Value (0-100)**
1	The Plunde app motivates me	
2	The Plunde app is easy and intuitive to use	
3	The goal that was set was right for you	
4	The tasks that were registered in the app were right for you	
5	The content of the reminders that Plunde gave you were right for you	
6	The reminders of your tasks appeared at the preset time	
7	It is simple and intuitive to answer that the reminder has been received	
8	The individual feedback from the supervisor was motivating	
9	The «my notes» function was useful	
10	The «statistics» function was useful	
11	The «video» function was useful	
12	The «knowledgebank» was useful	
13	The message function was useful	
	Is there anything you miss or that you think was particularly good?	

13: If you could choose, for how long would you prefer to be followed up through the Plunde app?

3 months ☐ 6 months ☐ 9 months ☐ 12 months ☐

14: How often would you find it useful to get feedback from your supervisor, if you were to be followed up for the time answered in question 13?

Weekly ☐ Biweekly ☐ Every third week ☐ Every month ☐

Do not want any feedback ☐

15: The first three months I would have found it motivating to get feedback from my supervisor:

Daily ☐ 2-4 time per week ☐ Weekly ☐ Biweekly ☐ Every month ☐
